# Association of Preoperative MRI-Derived Anatomical Parameters with Urinary Continence Recovery After Robot-Assisted Radical Prostatectomy

**DOI:** 10.3390/diagnostics16193203

**Published:** 2026-10-02

**Authors:** Deniz Öztürk Koçakgöl, Mehmet Eren Öztürk, Muhittin Atar, Hüseyin Koçakgöl, İbrahim Karabulut

**Affiliations:** 1 Department of Radiology, University of Health Sciences, Erzurum City Hospital, Erzurum 25240, Türkiye; dr.denizz@hotmail.com; 2 Department of Urology, University of Health Sciences, Erzurum City Hospital, Erzurum 25240, Türkiye; muhittinatar25@hotmail.com.tr (M.A.); hsynkocakgl@gmail.com (H.K.); karabulutibrahim36@gmail.com (İ.K.)

**Keywords:** robot-assisted radical prostatectomy, urinary incontinence, urinary continence, prostate magnetic resonance imaging

## Abstract

**Objectives:** This study evaluated the association of patient age and preoperative prostate MRI-derived anatomical parameters with postoperative urinary incontinence (UI) and urinary continence (UC) recovery after robot-assisted radical prostatectomy (RARP). **Methods:** We retrospectively included 118 patients who underwent RARP between 2022 and 2024 and had preoperative prostate MRI. Patient age and continence status at 1, 3, 6, and 12 months were obtained from medical records. Levator ani muscle (LAM), obturator internus muscle (OIM), and pubococcygeus muscle thicknesses were measured at their thickest points on axial and coronal T2-weighted images. Membranous urethral width (MUW), length (MUL), and angle (MUA) were measured, and prostate volume was calculated. UC was defined as no pad use or one safety pad per day (used for reassurance or for occasional minimal drops of urine), whereas UI was defined as the use of one or more pads per day that became wet because of urine leakage. Associations were evaluated using group comparisons, Kaplan–Meier analysis, and exploratory Cox and logistic regression models. **Results:** UC rates at 1, 3, 6, and 12 months were 32.2%, 54.2%, 73.7%, and 91.5%, respectively, while 8.5% of patients remained incontinent at 12 months. At 3 months, patients with UC differed significantly from those with UI in age, MUA, and LAM thickness. Comparisons among patients who achieved UC at different postoperative time points and those with UI at 12 months demonstrated significant differences in LAM thickness, MUL, and MUA (all *p* < 0.05). In multivariable Cox regression, higher MUA (hazard ratio [HR] 1.43 per 10°), thicker LAM (coronal HR 1.30 and axial HR 1.28 per mm), and younger age (HR 0.85 per 5 years) were associated with earlier continence recovery, whereas MUL was not. Prostate volume, MUW, and pubococcygeus muscle thickness were not associated with continence outcomes. **Conclusions:** Preoperative MRI-derived anatomical parameters, particularly MUA and LAM thickness, together with patient age, were associated with urinary continence recovery after RARP, and the associations of MUA and LAM thickness remained significant after multivariable adjustment in the time-to-event analysis. These exploratory findings are hypothesis-generating; clinical confounders could not be accounted for, and validation in prospective studies is required before these parameters can be considered for clinical use.

## 1. Introduction

Prostate cancer is the second most commonly diagnosed cancer among men worldwide and ranks fifth among the causes of cancer-related mortality. In recent years, a significant increase in the incidence of prostate cancer has been observed. The widespread use of prostate-specific antigen (PSA) screening, improved access to healthcare services, and increased public awareness may have contributed to this increase [1].

Robot-assisted radical prostatectomy (RARP) has become a well-established surgical option for localized prostate cancer. Despite its favorable oncological outcomes, urinary incontinence (UI) remains one of the most significant morbidities following radical prostatectomy, adversely affecting patients’ quality of life. In a meta-analysis, UI rates at 12 months after RARP ranged from 4% to 31% when continence was defined as no pad use and from 8% to 11% when continence was defined as no pad use or the use of a safety pad [2]. Another meta-analysis reported urinary continence (UC) rates of approximately 40–50% at 1 month, increasing to nearly 90% by 12 months [3].

Because UI following RARP represents a significant morbidity, an increasing number of studies have investigated factors that may predict UI development and the time to UC recovery. Identifying such factors may help guide preoperative patient counseling and inform expectations regarding the duration of UI. Preoperative prostate MRI-derived anatomical parameters, including pelvic floor muscle thickness, membranous urethral length (MUL), membranous urethral width (MUW), membranous urethral angle (MUA), and prostate volume, together with patient age, have been investigated in relation to the duration and severity of postoperative UI. Some studies have demonstrated that MUL and pelvic floor muscle thickness are significantly associated with the duration and severity of UI [4,5,6].

In this study, we evaluated the association of patient age and anatomical parameters derived from preoperative prostate MRI with postoperative UI and time to UC recovery in patients undergoing RARP.

## 2. Materials and Methods

The study was conducted in accordance with the principles of the Declaration of Helsinki and approved by the University of Health Sciences Ethics Committee (Approval No: 2025/03-71).

### 2.1. Patients

**Figure 1:**
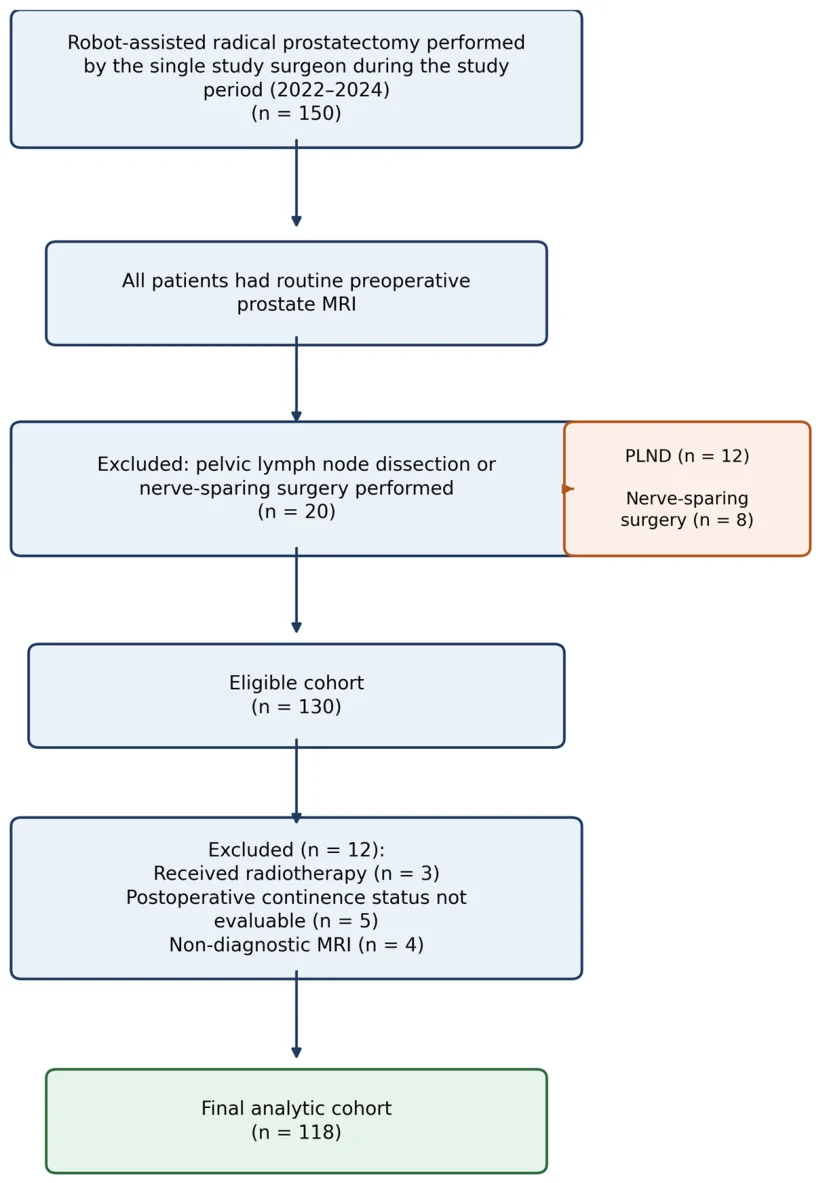
Patient screening and exclusion flow diagram. PLND: pelvic lymph node dissection.

Data from patients who underwent RARP at our hospital between 2022 and 2024 were retrospectively evaluated. To minimize variability in surgical technique, only patients who underwent RARP performed by a single surgeon and who had preoperative prostate MRI were included. Patients who underwent pelvic lymph node dissection (*n* = 12) or nerve-sparing surgery (*n* = 8) during the study period were excluded by design, as these procedures may influence continence recovery. In our practice, pelvic lymph node dissection is performed in patients with an expected risk of lymph node invasion of 7% or higher according to the nomogram developed by Gandaglia et al. [7], whereas nerve-sparing surgery is offered to young, sexually active patients with low-risk disease and an IIEF-5 score of 22–25; IIEF-5 is assessed routinely in our clinical practice. The clinical and oncological information used for these routine treatment decisions, including the nomogram score and IIEF-5, was available to the treating team at the time of surgical planning; however, because the present analysis was not planned at that time, these variables were not systematically extracted as structured variables into the retrospective research dataset and could therefore not be incorporated into the analyses. The study population therefore represents a selected subgroup of RARP patients. The patient screening and exclusion process is summarized in Figure 1. During the study period, 150 RARP procedures were performed by the study surgeon; after excluding those with pelvic lymph node dissection or nerve-sparing surgery, the initial cohort comprised 130 patients. Three patients who received radiotherapy, five patients whose postoperative urinary continence status could not be evaluated, and four patients whose initial MRI examination showed inadequate image quality (non-diagnostic due to motion artifacts) were subsequently excluded. The final analysis included 118 patients.

Patient age and UI data were obtained from medical records. UC was defined as the use of no pads or at most one safety pad per day, where a safety pad was defined as a pad used for reassurance or for occasional minimal drops of urine during strenuous activity, without the pad becoming soaked. UI was defined as the use of one or more pads per day that became wet because of urine leakage and required changing. These definitions are mutually exclusive. UI assessments were performed at 1, 3, 6, and 12 months.

### 2.2. Surgical Technique

All procedures were performed using conventional transperitoneal robot-assisted radical prostatectomy (RARP) with posterior dissection by a single surgeon with an experience of more than 100 RARP procedures before the study period; the Retzius-sparing approach was not used. Bladder neck sparing was performed, and the urethral stump was preserved during apical dissection; the length of the preserved stump was not measured. Cold scissors were used for both levator ani muscle dissection and distal urethral dissection, without electrocautery during these steps. No reconstruction, including fascial reconstruction, was performed before or after the vesicourethral anastomosis. The urethral catheter was removed 5–7 days after surgery.

After catheter removal, all patients received standardized verbal and written instructions on pelvic floor muscle (Kegel) exercises from the surgical team. Patients were instructed to identify the pelvic floor muscles, contract them for a count of 10 s, and relax for approximately 15 s between contractions; to perform 15 repetitions in each of three positions (lying, sitting, and standing), three times daily, for at least 6–8 weeks and to continue thereafter; and to contract the pelvic floor muscles during coughing, sneezing, or lifting. The exercises were self-directed, and no supervised physiotherapy was provided. At each postoperative follow-up visit, patients were asked whether they were performing the exercises, and their continence status was recorded; exercise adherence was not formally quantified. Written postoperative recommendations, including adequate hydration, avoidance of constipation and straining, and restriction of heavy lifting and activities placing pressure on the surgical area, were also provided.

### 2.3. MRI Protocol and Image Analysis

**Figure 2:**
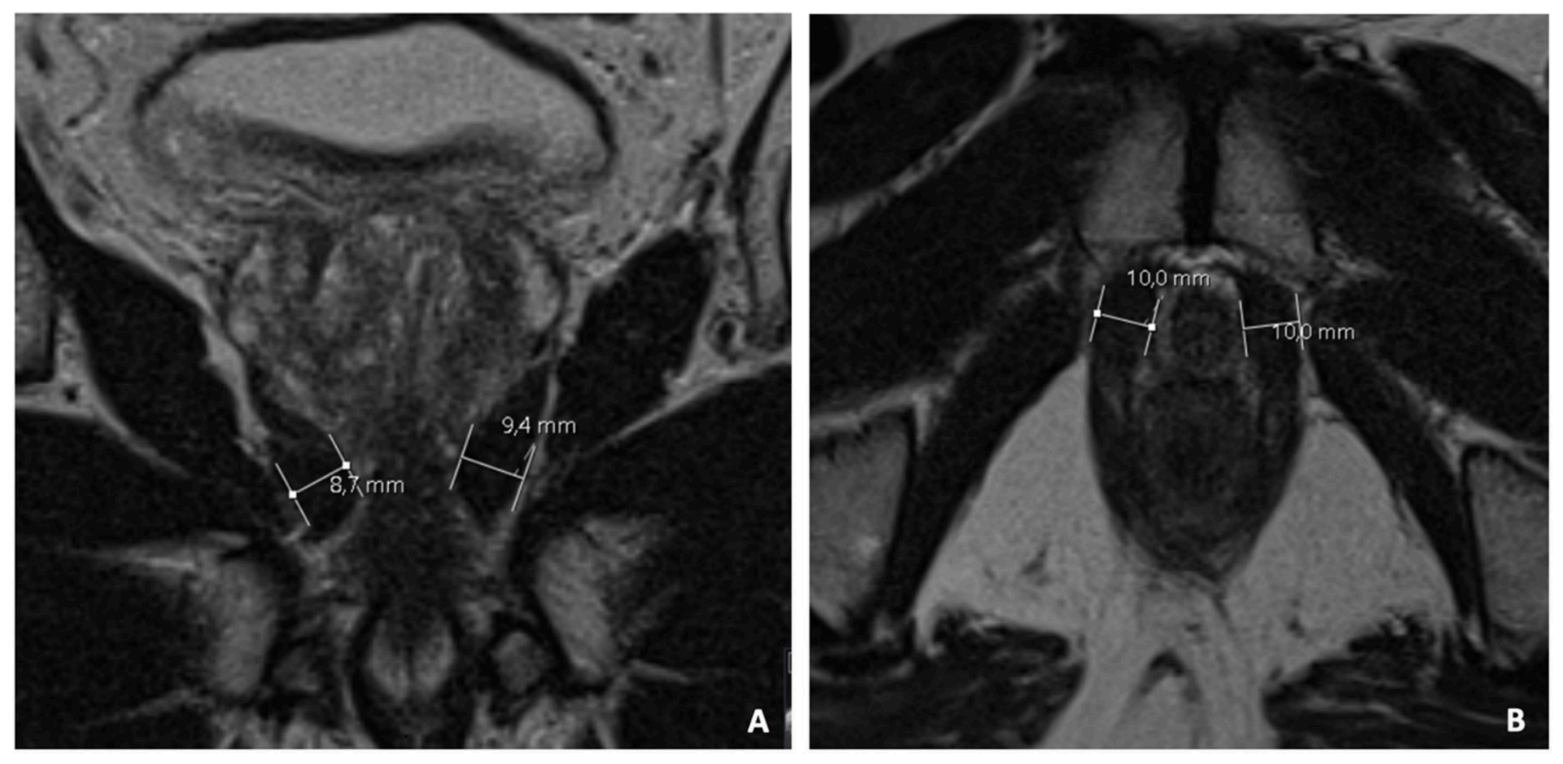
Coronal (**A**) and axial (**B**) T2-weighted MRI images showing the measurement of levator ani muscle thickness at the pelvic floor level, recorded at its maximum thickness.

**Figure 3:**
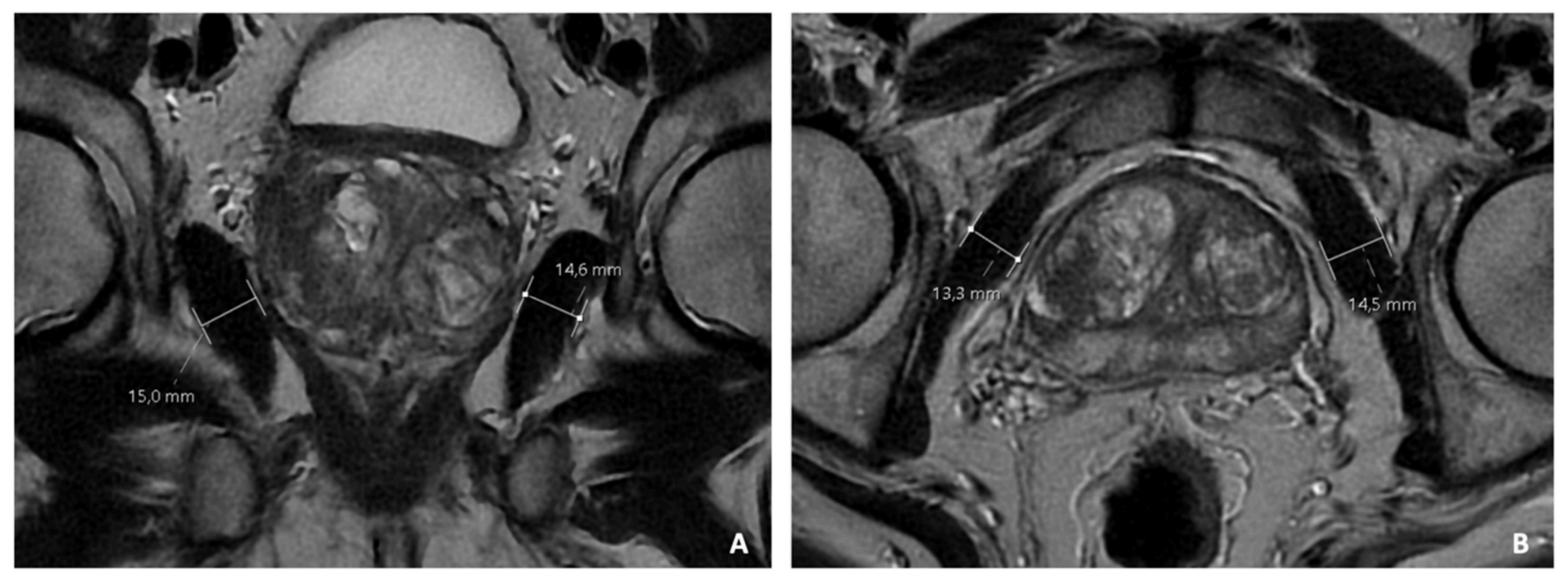
Coronal (**A**) and axial (**B**) T2-weighted MRI images showing the measurement of obturator internus muscle thickness, recorded at its maximum thickness.

**Figure 4:**
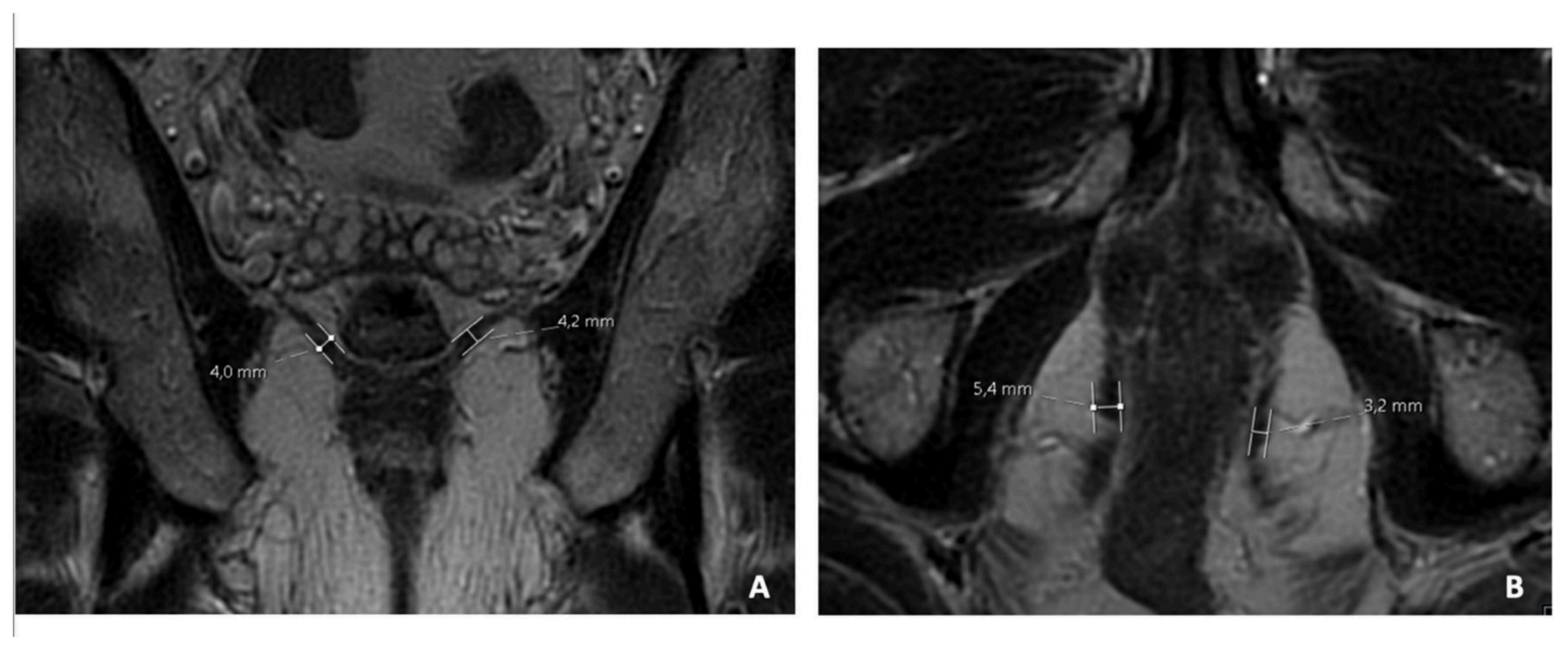
Coronal (**A**) and axial (**B**) T2-weighted MRI images showing the measurement of pubococcygeus muscle thickness, recorded at its maximum thickness.

**Figure 5:**
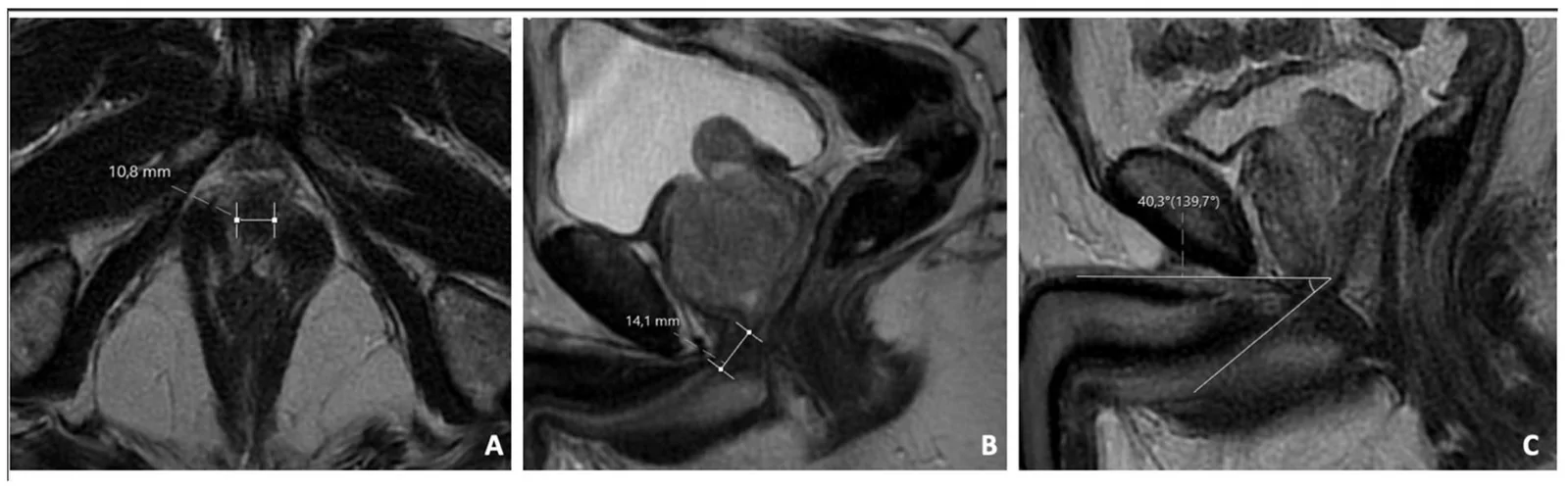
Measurement of membranous urethral width (MUW) on axial images at the widest section of the urethra just before entering the prostate (**A**). Measurement of membranous urethral length (MUL) on sagittal T2-weighted images from the point where the urethra enters the penile bulb to the prostate apex (**B**). Measurement of membranous urethral angle (MUA) on sagittal T2-weighted images, defined as the angle between the membranous urethra and a line drawn parallel to the axial plane from the inferior margin of the pubic symphysis (**C**). The value in parentheses (e.g., 139.7°) is the reported MUA, corresponding to 180° minus the measured acute angle (40.3°).

**Figure 6:**
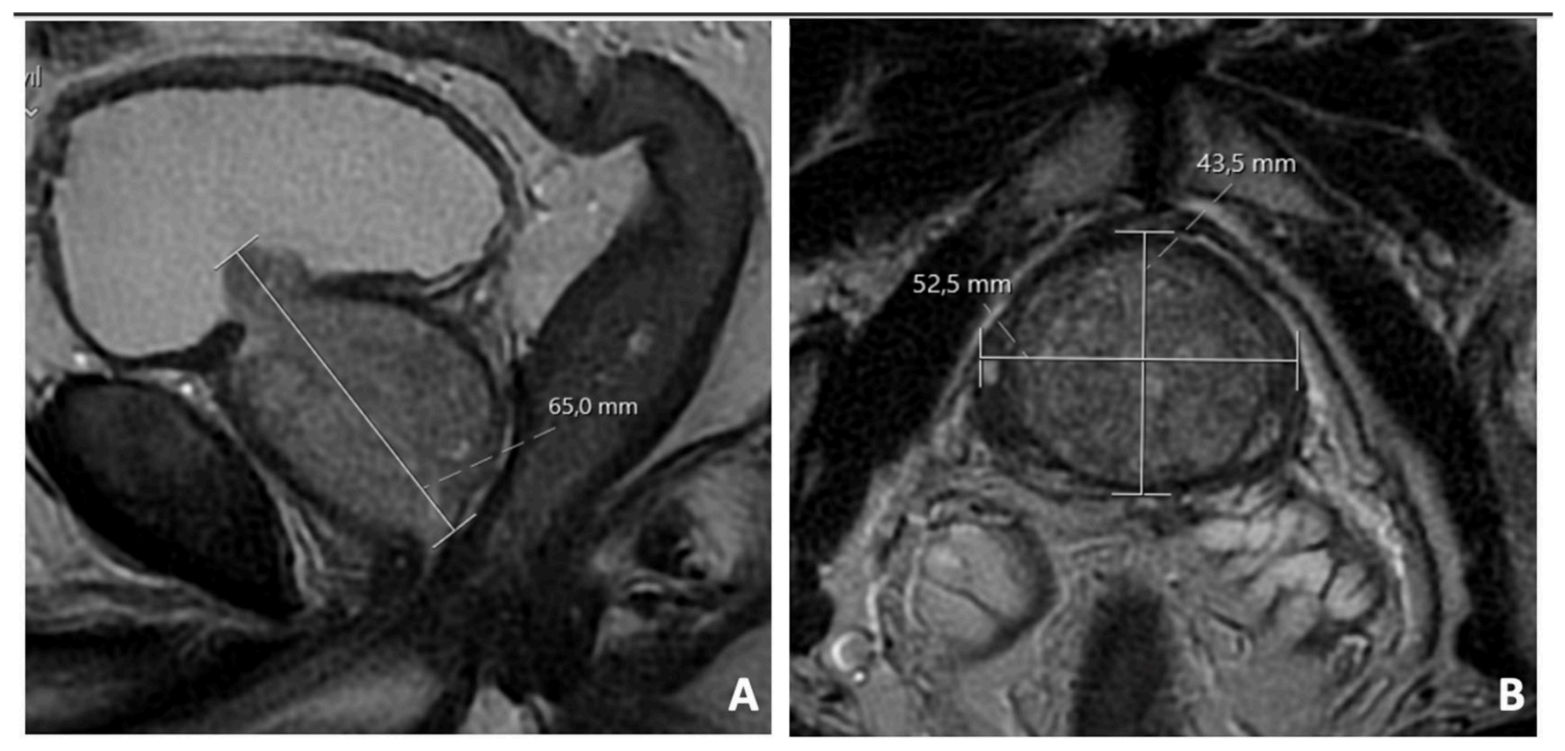
Sagittal T2-weighted MRI image demonstrating the measurement of prostate length (**A**). Axial T2-weighted MRI image demonstrating the measurement of prostate width and height (**B**). Prostate volume was calculated using the formula: width (cm) × height (cm) × length (cm) × π/6.

Prostate MRI examinations were performed using a 1.5 Tesla MR scanner (General Electric Signa Explorer, GE Medical Systems, Waukesha, WI, USA) with a pelvic coil. The protocol included T2-weighted sagittal, axial, and coronal images; T1-weighted axial images; diffusion-weighted images; and T1-weighted fat-suppressed dynamic contrast-enhanced images. All images were evaluated by two radiologists with 12 and 7 years of professional experience, and measurements were performed by consensus. The radiologists were blinded to postoperative continence outcomes; patient age was displayed in the picture archiving and communication system and was therefore not concealed. Other clinical data, such as PSA, were not analyzed in this study. On axial and coronal T2-weighted images, the thicknesses of the levator ani, obturator internus, and pubococcygeus muscles were measured at their thickest points, perpendicular to the muscle surface, on both sides, and the mean of the two sides was used (Figure 2, Figure 3 and Figure 4). Membranous urethral width (MUW) was measured at the widest section of the urethra just before entering the prostate on axial images (Figure 5). Membranous urethral length (MUL) was measured on sagittal T2-weighted images from the point where the urethra enters the penile bulb to the prostate apex (Figure 5). The membranous urethral angle (MUA) was measured on sagittal T2-weighted images as the angle between the membranous urethra and a line drawn parallel to the axial plane from the inferior margin of the pubic symphysis (Figure 5) [8]. MUA values are reported as the obtuse angle (180° minus the acute angle between the membranous urethra and the reference line), such that higher values indicate a less steeply inclined membranous urethra. Prostate volume was calculated using the formula: width (cm) × height (cm) × length (cm) × π/6 (Figure 6).

The examinations were performed for biopsy decision-making or preoperative evaluation, 2–8 weeks before RARP. Patients were asked to void before the examination and were examined in the supine position. The T2-weighted sequences were acquired with a repetition time (TR) of 5095 ms, an echo time (TE) of 124 ms, a slice thickness of 3 mm, an interslice gap of 0.5 mm, and a field of view of 220 mm in the sagittal plane, 200–220 mm in the coronal plane, and 220 mm in the axial plane. Diffusion-weighted images (b-values 50, 800, and 1500 s/mm^2^) were acquired with a TR of 2000 ms and a TE of 73 ms, and dynamic contrast-enhanced fat-suppressed T1-weighted images with a TR of 3 ms and a TE of 1.4 ms after intravenous administration of gadoteric acid (0.2 mL/kg). Examinations with inadequate image quality due to motion artifacts were considered non-diagnostic. Measurements were performed on the Sectra picture archiving and communication system (PACS). MUL and MUA were measured on the sagittal slice on which the urethra was best visualized.

### 2.4. Statistical Analysis

Descriptive statistics of the data (number, percentage, mean, standard deviation, median, 25th and 75th percentiles) were presented in this study. The assumption of normal distribution was assessed using the Shapiro–Wilk test, and homogeneity of variances was evaluated using Levene’s test. For comparisons of two independent groups, the Independent Samples *t*-test (with Welch’s correction where variances were unequal) was used when the normality assumption was met, whereas the Mann–Whitney U test was applied when the assumption was not satisfied. For comparisons of three or more independent groups, ANOVA (Welch’s ANOVA where variances were unequal) was used when the data were normally distributed, and the Kruskal–Wallis test was used when normal distribution was not present. Post hoc Bonferroni or Tamhane tests, selected according to the homogeneity of variances, were used following ANOVA, and Bonferroni-adjusted pairwise comparisons were used following the Kruskal–Wallis test, to identify the specific group or groups responsible for the differences. The Cochran’s Q test was used to assess the association between dependent categorical variables, and pairwise comparisons were performed using the McNemar test with Holm–Bonferroni correction. Correlations between MUA and the other continuous parameters were assessed using Spearman’s rank correlation coefficient.

To address the limitations of comparing discrete groups, additional exploratory analyses were performed. Time to continence was defined as the first follow-up visit (1, 3, 6, or 12 months) at which the patient was continent; patients with UI at 12 months were censored at 12 months. Because continence was assessed only at these fixed visits, the exact time of transition to continence was unknown, and the resulting data more accurately represent interval-censored observations. The Cox model was therefore fitted using the visit time as an approximation of the true event time, which may introduce some imprecision. Cumulative continence was estimated using the Kaplan–Meier method, and groups defined by the median of each parameter were compared with the log-rank test. Associations of continuous parameters with time to continence were assessed with Cox proportional hazards regression, first univariably and then in a multivariable model including age, MUA, MUL, and coronal and axial LAM thickness (108 events; 21.6 events per variable). Multicollinearity among the variables in the multivariable models was assessed using variance inflation factors. Hazard ratios (HR) are reported with 95% confidence intervals (CI) per 5 years of age, 10° of MUA, 10 cm^3^ of prostate volume, and 1 mm of the remaining measurements; an HR > 1 indicates faster continence recovery. For the 11 parameters examined in univariable Cox regression, a Bonferroni-adjusted significance level of 0.0045 was applied to account for multiple comparisons, and the discrimination of the multivariable Cox model was summarized with Harrell’s concordance index. The proportional hazards assumption was assessed using Schoenfeld residuals. As a sensitivity analysis accounting for repeated assessments, generalized estimating equations (GEE; binomial family, logit link, exchangeable correlation) were used to model UI at all four time points, with the assessment time point (1, 3, 6, or 12 months) included as a categorical covariate together with age, MUA, MUL, and coronal and axial LAM thickness. At 3 months (54 patients with UI), an exploratory multivariable logistic regression including age, MUA, and coronal and axial LAM thickness was fitted (13.5 events per variable). Results of the logistic models are reported as odds ratios (OR) with 95% CI. Discrimination was assessed using the area under the receiver operating characteristic curve (AUC), with 95% CI obtained by bootstrap percentile resampling (1000 replicates). Bootstrap resampling (1000 replicates) was also used to estimate the optimism-corrected AUC, calibration slope, and Brier score and to derive a bias-corrected calibration curve (locally weighted regression; Supplementary Figure S1), and the added value of the MRI parameters over age was tested with a likelihood-ratio test. Because only 10 patients had UI at 12 months, multivariable modeling was not attempted at this time point, and univariable associations were estimated with Firth’s penalized logistic regression. These analyses were not prespecified. Variables were selected on the basis of the primary analyses and previous literature; clinical covariates such as body mass index (BMI), diabetes, PSA, and tumor stage were not available. Descriptive statistics and group comparisons were performed using IBM Statistical Package for the Social Sciences (SPSS) for Windows, version 27 (SPSS Inc., Chicago, IL, USA). Correlation, time-to-event, regression, and validation analyses were performed using Python 3.12 (SciPy 1.17, lifelines 0.30, statsmodels 0.15, and scikit-learn 1.8); Firth’s penalized logistic regression was implemented with custom code. A two-sided *p*-value of <0.05 was considered statistically significant.

## 3. Results

### 3.1. Patient Characteristics and Continence Rates

Descriptive statistics for the clinical characteristics of the patients are presented in Table 1. Cumulative UC rates at 1, 3, 6, and 12 months were 32.2%, 54.2%, 73.7%, and 91.5%, respectively. Overall, 32.2% of patients achieved continence at 1 month, 22.0% at 3 months, 19.5% at 6 months, and 17.8% at 12 months, whereas 8.5% remained incontinent at 12 months. The proportion of continent patients increased significantly over time (Cochran’s Q = 140.1, df = 3, *p* < 0.001; all pairwise McNemar comparisons with Holm–Bonferroni correction *p* < 0.001).

Median, mean, and range values for age, prostate volume, MUL, MUW, MUA, LAM thickness in coronal and axial planes, OIM thickness in coronal and axial planes, and pubococcygeus muscle thickness in coronal and axial planes are presented in Table 1.

### 3.2. Comparisons According to Continence Status

The distribution of measurements according to the 3rd-month UC and UI groups is presented in Table 2, and comparisons were performed using the Independent Samples t-test and Mann–Whitney U test. The analyses revealed statistically significant differences in age, MUA, and both coronal and axial measurements of LAM thickness between the 3rd-month UC and UI groups (*p* < 0.05): patients in the UI group were older, whereas the UC group had higher MUA and LAM thickness values. No statistically significant differences were observed in the other measurements according to 3rd-month incontinence status (*p* > 0.05).

The distribution of measurements according to the study groups is presented in Table 3, and ANOVA and Kruskal–Wallis tests were used to compare the groups. The analyses revealed statistically significant differences in MUL, MUA, and both coronal and axial measurements of LAM thickness among the study groups (*p* < 0.05).

For MUL, post hoc Bonferroni comparison showed significantly higher values in the 6th-month UC group than in the 12th-month UI group (*p* = 0.027). For MUA, post hoc Bonferroni comparisons showed significantly higher values in the 1st-month and 3rd-month UC groups than in the 12th-month UI group (both *p* < 0.001). For coronal LAM thickness, post hoc Tamhane comparisons revealed that the 1st-month UC group had significantly greater values than the 3rd-month UC, 6th-month UC, 12th-month UC, and 12th-month UI groups (*p* < 0.001, *p* = 0.003, *p* < 0.001, and *p* < 0.001, respectively). For axial LAM thickness, post hoc Tamhane comparisons further demonstrated that the 1st-month UC group had significantly greater values than the 3rd-month UC, 12th-month UC, and 12th-month UI groups, and that the 3rd-month UC group had significantly greater values than the 12th-month UC group (*p* = 0.029, *p* < 0.001, *p* = 0.013, and *p* = 0.003, respectively). No other significant differences were observed among the study groups (*p* > 0.05).

### 3.3. Time to Continence and Multivariable Analyses

**Figure 7:**
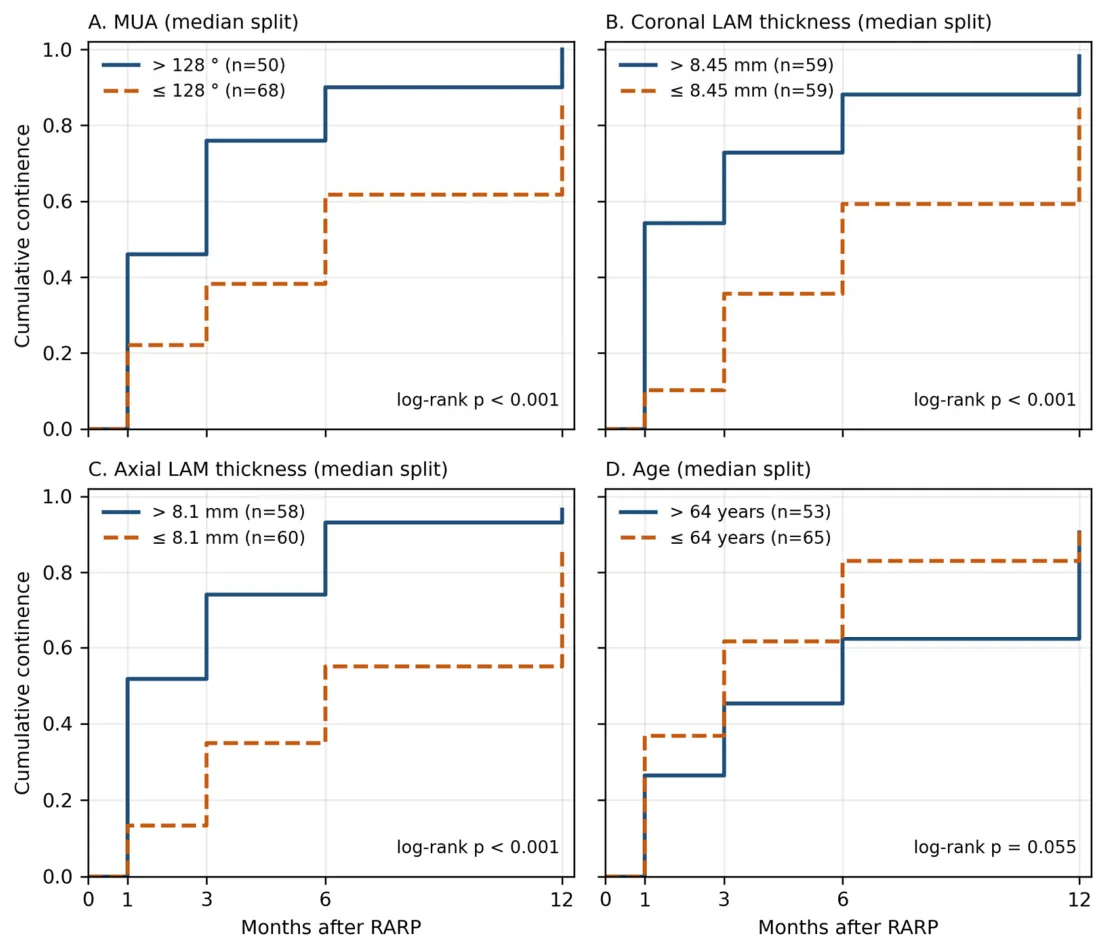
Cumulative continence after RARP according to the median of (**A**) MUA, (**B**) coronal LAM thickness, (**C**) axial LAM thickness, and (**D**) age. Kaplan–Meier estimates at the 1-, 3-, 6-, and 12-month assessments; patients with UI at 12 months were censored at 12 months. *p* values are from the log-rank test.

Cumulative continence over time according to the median of each parameter is shown in Figure 7. Patients with MUA above the median (128°) had higher cumulative continence at 1, 3, and 6 months (46%, 76%, and 90%) than those below the median (22%, 38%, and 62%; log-rank *p* < 0.001). Similar differences were observed for coronal LAM thickness (54%, 73%, and 88% vs. 10%, 36%, and 59%; *p* < 0.001) and axial LAM thickness (52%, 74%, and 93% vs. 13%, 35%, and 55%; *p* < 0.001), whereas no significant differences were observed for MUL (*p* = 0.466) or age (*p* = 0.055).

In univariable Cox regression (Table 4), higher MUA, coronal and axial LAM thickness, and younger age were associated with earlier continence recovery. Coronal OIM thickness showed a weak association (HR 1.09 per mm, *p* = 0.033), whereas prostate volume, MUW, MUL, axial OIM thickness, and pubococcygeus muscle thickness were not associated. After Bonferroni correction for the 11 parameters (α = 0.0045), only MUA and LAM thickness remained significant. In the multivariable model, MUA (HR 1.43 per 10°), coronal LAM (HR 1.30 per mm), axial LAM (HR 1.28 per mm), and age (HR 0.85 per 5 years) remained associated with time to continence, whereas MUL did not (concordance index 0.81). Variance inflation factors were ≤1.50 for all variables in the multivariable models, indicating no relevant collinearity. The proportional hazards assumption was not violated for most variables, with a borderline deviation for coronal LAM thickness (*p* = 0.040).

In the generalized estimating equation (GEE) model including all four assessments, higher MUA (OR for UI 0.57 per 10°, 95% CI 0.40–0.82; *p* = 0.002) and coronal (OR 0.50 per mm, 0.36–0.70) and axial (OR 0.53, 0.39–0.73) LAM thickness were associated with lower odds of UI, whereas age (OR 1.32 per 5 years, 0.96–1.81; *p* = 0.091) and MUL (OR 1.08, 0.92–1.27; *p* = 0.342) were not.

At 3 months, in the multivariable logistic model (Table 5), MUA, coronal LAM, and axial LAM thickness were associated with UI, whereas age was not (*p* = 0.077). The model including age and the MRI parameters had an AUC of 0.85 (95% CI 0.77–0.91; optimism-corrected 0.82; calibration slope 0.87), compared with 0.62 for age alone (likelihood-ratio test for the added MRI parameters, χ^2^ = 44.9, df = 3, *p* < 0.001). Individual AUCs were 0.76 for coronal and axial LAM thickness, 0.70 for MUA, 0.62 for age, and 0.55 for MUL. The bias-corrected calibration curve was close to the line of identity (Supplementary Figure S1); the bootstrap-corrected calibration slope of 0.87 indicated mild overfitting, and the corrected Brier score was 0.176 (0.248 for a model without predictors). Among the 10 patients with UI at 12 months (Table 5), univariable Firth regression showed associations with MUA, LAM thickness, and MUL but not age; given the small number of events, these estimates are imprecise, and no multivariable model was fitted.

### 3.4. Correlations Among MRI Parameters

In an exploratory analysis, MUA showed weak, non-significant correlations with age (ρ = 0.08, *p* = 0.384), prostate volume (ρ = 0.06, *p* = 0.495), MUL (ρ = 0.02, *p* = 0.871), MUW (ρ = 0.07, *p* = 0.484), coronal LAM thickness (ρ = 0.08, *p* = 0.396), and axial LAM thickness (ρ = 0.18, *p* = 0.058).

## 4. Discussion

In this study, patient age, MUA, and LAM thickness were significantly associated with continence status at 3 months after RARP. Comparisons among the study groups further demonstrated significant differences in MUL, MUA, and LAM thickness according to the timing of continence recovery. These findings suggest that preoperative prostate MRI-derived anatomical parameters, together with patient age, may be associated with postoperative continence recovery following RARP. The associations of MUA and LAM thickness persisted in time-to-event and multivariable analyses, whereas MUL was associated only with urinary incontinence at 12 months in a univariable analysis.

Continence rates at 1, 3, 6, and 12 months (32.2%, 54.2%, 73.7%, and 91.5%, respectively) were consistent with previously reported outcomes [5]. Increasing age has been identified as an important risk factor for postoperative urinary incontinence in several studies [9]. Consistent with this, a significant difference in age was observed between the UC and UI groups at 3 months, in line with the findings of Al-Kalailah et al. [10], who reported advanced age as an independent risk factor for incontinence at 3 months, and Greco et al. [11], who reported similar findings at 6 months. However, no significant difference in age was observed among the UC groups at 1, 3, 6, and 12 months and the UI group at 12 months, which may be attributable to the limited sample size in some subgroups. Taken together, these findings suggest that the influence of age on postoperative UC recovery requires further validation in larger studies. In multivariable analyses, age remained associated with time to continence in Cox regression (HR 0.85 per 5 years) but not with UI at 3 months after adjustment for MRI parameters (*p* = 0.077), and these results require confirmation in larger cohorts.

Prostate volume was not significantly associated with the time to UC recovery in our study, consistent with prior findings. Liu et al. reported that prostate volume was associated with immediate UC in the early postoperative period but had no significant effect on UC at 1 or 3 months [12]. Similarly, Al-Kalailah et al. found a significant relationship between UC and prostate volume only in the early postoperative period, with no association observed at 1 or 3 months [10]. As early postoperative continence data were not available in our study, direct comparison with studies evaluating immediate postoperative continence was not possible.

An increase in MUL has been associated with shorter time to UC recovery in the literature [13]. Wang et al. reported that larger preoperative three-dimensional membranous urethral volume and length parameters were associated with earlier continence recovery after RARP, although the membranous urethral length parameters did not differ significantly between continent and incontinent patients at 3 months, consistent with our 3rd-month findings [14]. In our study, however, MUL differed significantly between the 6th-month UC group and the 12th-month UI group. In time-to-event analysis, MUL was not associated with the speed of continence recovery (HR 1.04 per mm, *p* = 0.252). In a univariable analysis limited to the 12-month time point (10 incontinent vs. 108 continent patients), MUL was associated with urinary incontinence at 12 months (OR 0.65 per mm, 95% CI 0.45–0.94); because this analysis was univariable, it cannot establish an independent association, and the finding should be interpreted as hypothesis-generating rather than confirmatory. This is in line with the meta-analysis by Mac Curtain et al., which reported a significant association between increased MUL and reduced UI at 12 months [13]. In contrast, Veccia et al. found no significant difference in MUL between the UC and UI groups at 12 months [15]. Collectively, the available evidence suggests that the association between MUL and postoperative UC recovery remains inconclusive.

Consistent with our findings, Veccia et al. reported no significant association between MUW and UC [15]. Likewise, Kim et al. found no significant association between MUW and continence status at 3 months after RARP [4]. Collectively, these findings suggest that the current evidence does not support a consistent association between MUW and postoperative UC, and further studies with larger sample sizes and standardized measurement methods are warranted to better define its potential role in continence recovery.

In the present study, the MUA measurement method described by Sauer et al. was adopted to ensure standardized measurements [8]. Sauer et al. investigated this same parameter but found no significant association between preoperative MUA and postoperative continence [8]; to our knowledge, a significant association between preoperative MUA and postoperative UC after RARP has not been previously reported. We observed significantly higher MUA values in the UC group than in the UI group at 3 months after surgery. Furthermore, MUA was significantly higher in the 1st- and 3rd-month UC groups than in the 12th-month UI group. These findings suggest a potential association between preoperative MUA, when measured using a standardized method, and postoperative continence recovery following RARP. In multivariable analyses, MUA remained associated with continence recovery after adjustment for age, MUL, and LAM thickness (HR 1.43 per 10°); however, adjustment for clinical confounders was not possible, and its independent value remains to be established. Although the underlying mechanism cannot be established from the present data, several explanations may be considered. In our measurement convention, a larger MUA corresponds to a smaller inclination of the membranous urethra relative to the axial plane through the inferior margin of the pubic symphysis. Such an orientation may reflect a urethral course in which the sphincter complex and the future vesicourethral anastomosis remain well aligned within the support of the pelvic floor, potentially favoring urethral coaptation and pressure transmission after prostate removal. Conversely, a steeper inclination might predispose to greater angulation or traction at the anastomotic site once the prostate is removed. MUA may also reflect broader pelvic or apical anatomy not captured by the other parameters; in our cohort, MUA showed no statistically significant correlation with age, prostate volume, MUL, MUW, or LAM thickness, suggesting that it does not simply mirror these variables, consistent with its persistent association after adjustment for these variables in multivariable models. These explanations are speculative and hypothesis-generating; whether MUA plays an independent or causal role in continence recovery requires validation in prospective studies that include functional assessments (e.g., pad-weight testing or validated symptom questionnaires) and are adjusted for clinical and surgical confounders.

LAM thickness differed significantly between the UC and UI groups at 3 months. Post hoc comparisons further showed that the 1-month UC group had significantly greater coronal LAM thickness than the 3-, 6-, and 12-month UC groups and the 12-month UI group, and significantly greater axial LAM thickness than the 3- and 12-month UC groups and the 12-month UI group; axial LAM thickness was also significantly greater in the 3-month UC group than in the 12-month UC group [15,16]. These findings suggest that greater preoperative LAM thickness may be associated with earlier postoperative continence recovery. This association was confirmed in Kaplan–Meier, Cox, and GEE analyses and persisted after adjustment for age and MUA, and LAM thickness showed the best discrimination among the individual parameters (AUC 0.76 for UI at 3 months). However, as clinical confounders could not be accounted for, this association should be interpreted cautiously and requires validation in larger prospective studies.

Although previous studies have suggested that increased OIM thickness may contribute to continence recovery, with significant differences reported between the UC and UI groups [6,17], OIM thickness was not significantly associated with continence status in the group comparisons in our study. Our findings are also consistent with those of Li et al., who found no association between OIM thickness and early UC, although their evaluation was performed during the first postoperative week, limiting direct comparison with our findings [18]. Overall, the number of studies investigating the role of OIM thickness in continence recovery after RARP remains limited, and further research is needed to clarify its clinical relevance. In time-to-event analysis, coronal OIM thickness showed a weak univariable association with earlier continence recovery (HR 1.09 per mm, *p* = 0.033) that did not withstand correction for multiple comparisons and was not observed for axial thickness (*p* = 0.147); this finding should be interpreted with caution.

The pubococcygeus muscle is a component of both the pelvic floor and the LAM complex. Although numerous studies have investigated the association between LAM thickness and continence recovery after RARP, Hashimoto et al. were the only authors to specifically evaluate the pubococcygeus muscle [6]. They reported significantly greater pubococcygeus muscle thickness in the UC group than in the UI group. In contrast, no significant difference in pubococcygeus muscle thickness was observed between the UC and UI groups in our study. This discrepancy may reflect differences in patient characteristics, measurement techniques, or sample size. Given the limited available evidence, the role of the pubococcygeus muscle and other components of the LAM complex in postoperative continence recovery warrants further investigation.

This study has several limitations. First, its retrospective design carries an inherent risk of selection and information bias. Second, the relatively small sample size, particularly within some subgroup analyses, may have limited the statistical power to detect associations between anatomical parameters and postoperative continence outcomes. Third, although exploratory multivariable analyses were performed, they were not prespecified, were limited to age and MRI parameters, and could not adjust for clinical confounders such as BMI, diabetes, baseline urinary function, prior transurethral resection, PSA, or tumor stage, which were not available. The models were fitted in 118 patients with only 10 events at 12 months, variable selection was not repeated within the bootstrap validation, and no external validation was performed, so performance estimates may be optimistic. No minimal clinically important differences or clinical cut-off values were defined, and sensitivity, specificity, and decision-curve analyses were not performed, because cut-offs derived in the same small cohort would be unstable and optimistic. Therefore, the observed associations, particularly those involving MUA and LAM thickness, should be interpreted as preliminary associations rather than evidence of an independent role in continence recovery, and their clinical applicability should be interpreted with caution.

Fourth, the absence of first-week postoperative continence data precluded direct comparison with studies evaluating very early continence recovery. Fifth, pelvic floor muscle training consisted of standardized instructions without supervised physiotherapy, and adherence was not formally quantified, which may have influenced continence recovery independently of the MRI parameters. Sixth, continence was assessed based on daily pad use recorded at follow-up visits rather than validated instruments such as the 24-h pad test, pad weight, or the International Consultation on Incontinence Questionnaire–Urinary Incontinence Short Form (ICIQ-UI SF), and incontinence subtype was not recorded. Seventh, the cohort was highly selected. Patients who underwent nerve-sparing surgery (*n* = 8) or pelvic lymph node dissection (*n* = 12) were excluded by design to minimize variability in surgical technique. These exclusions were based on routine clinical and oncological decision-making, including an expected lymph-node invasion risk threshold for PLND and predefined clinical criteria, including IIEF-5 (routinely assessed in our practice), for nerve-sparing surgery. However, individual-level nomogram scores and IIEF-5 scores, as well as detailed oncological risk classification and clinical and pathological stage and grade, were not systematically extracted as structured variables into the research dataset because the present analysis was not planned when these treatment decisions were made. This selection process introduces the possibility of selection bias, as the included patients may differ systematically from excluded patients in age, functional characteristics, and oncological risk. Therefore, the findings may not be generalizable to the broader RARP population, particularly patients undergoing nerve-sparing surgery, pelvic lymph node dissection, other technical variants of RARP, or treatment at other institutions. Eighth, MRI measurements were performed by consensus of two radiologists, which precluded the assessment of interobserver agreement, and measurement reproducibility (intra- and interobserver) was not evaluated; this is a significant limitation, particularly for manual measurements such as MUA and LAM thickness. In the absence of intraclass correlation coefficients, Bland–Altman analyses, or other repeatability estimates, these MRI-derived measurements should not be considered sufficiently reproducible for routine clinical implementation, and the reported associations require confirmation in studies using blinded, independent readers and formal reproducibility assessments. In addition, the radiologists could not be blinded to patient age. Taken together, the absence of clinical and oncological covariates and the lack of formal MRI measurement reproducibility represent important limitations that may affect the validity and generalizability of the reported associations. Finally, methodological differences across studies may have limited the comparability and generalizability of our findings. Future prospective studies with larger cohorts, longer follow-up, robust multivariable statistical models incorporating relevant clinical and surgical covariates, and independent, blinded MRI measurements with reproducibility assessment are needed to validate these findings and determine whether these MRI-derived parameters have an independent association with continence recovery after RARP.

## 5. Conclusions

Our findings show that preoperative MRI-derived anatomical parameters, particularly MUA and LAM thickness, together with patient age, were associated with urinary continence recovery after RARP, and the associations of MUA and LAM thickness remained significant after multivariable adjustment in the time-to-event analysis. MUL was associated with urinary incontinence at 12 months in a univariable analysis (10 incontinent vs. 108 continent patients) and cannot be considered an independent predictor, whereas prostate volume, MUW, and pubococcygeus muscle thickness were not associated with continence outcomes. These results are exploratory and hypothesis-generating: the analyses were not prespecified, MRI measurement reproducibility was not assessed, clinical confounders were not available, and no external validation was performed. Accordingly, these parameters cannot currently be regarded as clinically applicable imaging biomarkers or used for individualized counseling; prospective studies with larger cohorts, validated continence measures, reproducible MRI measurements, and adjustment for clinical and surgical covariates are required.

## Figures and Tables

**Table 1:** Distribution of patients’ clinical characteristics and prostate MRI measurements.

		*n*	%
1 month	UC	38	32.2
	UI	80	67.8
3 months	UC	64	54.2
	UI	54	45.8
6 months	UC	87	73.7
	UI	31	26.3
12 months	UC	108	91.5
	UI	10	8.5
Study Groups	UC at 1 month	38	32.2
	UC at 3 months	26	22.0
	UC at 6 months	23	19.5
	UC at 12 months	21	17.8
	UI at 12 months	10	8.5
Parameters	Min-Max	Mean ± SD (Median)	
Age	44–79	63.77 ± 6.75 (64)	
Prostate volume (cm^3^)	17–294	59.8 ± 41 (48.5)	
MUL (mm)	4.6–16.9	9.77 ± 2.39 (9.6)	
MUW (mm)	7.9–16.6	10.87 ± 1.67 (10.7)	
MUA (^o^)	100–163	128.58 ± 12.45 (128)	
LAM coronal(mm)	5.4–12.1	8.49 ± 1.54 (8.45)	
LAM axial (mm)	5–13.7	8.33 ± 1.46 (8.1)	
OIM coronal (mm)	11.9–22.5	17.01 ± 2.4 (17.1)	
OIM axial (mm)	11.9–23.8	18.01 ± 2.54 (18.3)	
Pubococcygeus muscle coronal (mm)	1.5–6.1	3.65 ± 0.85 (3.6)	
Pubococcygeus muscle axial (mm)	2–6	3.65 ± 0.83 (3.6)	

UC: urinary continence, UI: urinary incontinence, MUL: membranous urethral length, MUW: membranous urethral width, MUA: membranous urethral angle, LAM: levator ani muscle, OIM: obturator internus muscle.

**Table 2:** Distribution and comparison of measurements according to the 3rd-month urinary continence (UC) and urinary incontinence (UI) groups.

Parameter	Group	Mean ± SD	Median	Test Statistic	*p*
			(25th–75th Percentile)		
Age	UC	62.59 ± 5.86	63 (59.5–67)	−2.051 †	0.043 *
	UI	65.17 ± 7.49	66 (60–71)		
Prostate volume	UC	59.63 ± 44.69	46.5 (37.5–69.5)	−0.519	0.604
	UI	60.00 ± 36.56	49.5 (39–65)		
MUL	UC	9.56 ± 2.13	9.35 (8–10.7)	−0.935	0.35
	UI	10.02 ± 2.67	9.75 (8.2–11.8)		
MUW	UC	11.02 ± 1.66	11 (10–11.85)	−1.205	0.228
	UI	10.70 ± 1.69	10.2 (9.7–11.8)		
MUA	UC	132.42 ± 12.79	130 (123–139)	3.865 †	<0.001 *
	UI	124.02 ± 10.42	124.5 (118–128)		
LAM Coronal	UC	9.13 ± 1.37	8.9 (8.2–10.05)	−4.891	<0.001 *
	UI	7.74 ± 1.39	7.35 (6.6–8.7)		
LAM Axial	UC	8.90 ± 1.30	8.75 (7.9–9.75)	−4.839	<0.001 *
	UI	7.64 ± 1.34	7.5 (7–8.3)		
OIM Coronal	UC	17.15 ± 2.51	17.1 (15.25–19.05)	0.676 †	0.501
	UI	16.84 ± 2.28	17.1 (14.7–18.3)		
OIM Axial	UC	18.05 ± 2.68	18.7 (16.2–20.05)	0.170 †	0.866
	UI	17.97 ± 2.38	18.1 (16.3–19.7)		
Pubococcygeus muscle coronal	UC	3.73 ± 0.78	3.6 (3.15–4.2)	−0.749	0.454
	UI	3.55 ± 0.92	3.55 (3.1–4.1)		
Pubococcygeus muscle axial	UC	3.67 ± 0.81	3.6 (3–4.15)	−0.241	0.81
	UI	3.62 ± 0.87	3.6 (3–4.2)		

* *p* < 0.05 and † Independent Samples *t*-test; otherwise, Mann–Whitney U test.UC: urinary continence, UI: urinary incontinence, MUL: membranous urethral length, MUW: membranous urethral width, MUA: membranous urethral angle, LAM: levator ani muscle, OIM: obturator internus muscle.

**Table 3:** Distribution and comparison of measurements according to the study groups.

		Mean ± SD	Median (25th–75th Percentile)	Test Statistic	*p*
Age	UC at 1 month	62.47 ± 6.37	63 (59–67)	8.417	0.077
	UC at 3 months	62.77 ± 5.14	63 (60–67)		
	UC at 6 months	63.22 ± 6.93	64 (59–68)		
	UC at 12 months	66.52 ± 7.14	68 (64–72)		
	UI at 12 months	66.8 ± 9.08	66.5 (59–76)		
Prostate volume	UC at 1 month	63.24 ± 49.69	48.5 (32–75)	1.300	0.861
	UC at 3 months	54.35 ± 36.48	44.5 (38–64)		
	UC at 6 months	54.35 ± 29.62	49 (31–63)		
	UC at 12 months	67.29 ± 45.72	50 (44–74)		
	UI at 12 months	57.7 ± 29.47	50.5 (36–73)		
MUL	UC at 1 month	9.63 ± 2.14	9.7 (8.1–10.9)	10.265	0.036 *
	UC at 3 months	9.46 ± 2.14	9.05 (8–10.5)		
	UC at 6 months	10.94 ± 2.86	10.9 (8.9–12.3)		
	UC at 12 months	9.96 ± 2.08	9.6 (8.4–10.8)		
	UI at 12 months	8.06 ± 2.44	7.4 (6.3–9.7)		
MUW	UC at 1 month	11.01 ± 1.54	10.9 (10.1–11.9)	1.838	0.766
	UC at 3 months	11.03 ± 1.86	11 (9.8–11.6)		
	UC at 6 months	10.67 ± 1.71	10.1 (9.3–12)		
	UC at 12 months	10.89 ± 1.87	10.3 (9.8–11.8)		
	UI at 12 months	10.41 ± 1.31	10.35 (9.7–11)		
MUA	UC at 1 month	131.58 ± 12.51	130 (123–138)	6.068 ‡	<0.001 *
	UC at 3 months	133.65 ± 13.35	131.5 (123–140)		
	UC at 6 months	126.48 ± 10.8	126 (119–132)		
	UC at 12 months	125.76 ± 8.44	125 (120–128)		
	UI at 12 months	114.7 ± 8.74	111.5 (109–120)		
LAM Coronal	UC at 1 month	9.72 ± 1.34	9.7 (8.7–10.8)	14.011 ‡	<0.001 *
	UC at 3 months	8.28 ± 0.88	8.3 (7.8–8.9)		
	UC at 6 months	8.21 ± 1.49	7.7 (7–9.5)		
	UC at 12 months	7.57 ± 1.12	7.4 (6.6–8.5)		
	UI at 12 months	7.03 ± 1.38	6.7 (6.1–8.2)		
LAM Axial	UC at 1 month	9.28 ± 1.36	9.3 (8.2–10.2)	12.800 ‡	<0.001 *
	UC at 3 months	8.36 ± 1.01	8.15 (7.7–9)		
	UC at 6 months	8.28 ± 1.32	8.1 (7.5–9.1)		
	UC at 12 months	7.29 ± 0.86	7.3 (7–7.8)		
	UI at 12 months	6.91 ± 1.68	7.15 (5.4–8)		
OIM Coronal	UC at 1 month	17.25 ± 2.72	17.3 (15.2–19.4)	1.793 ‡	0.143
	UC at 3 months	16.99 ± 2.21	17 (15.5–18.4)		
	UC at 6 months	17.62 ± 2.28	17.6 (16.4–18.9)		
	UC at 12 months	16.64 ± 2.09	16.4 (14.7–17.9)		
	UI at 12 months	15.49 ± 2.15	16.4 (13.3–16.7)		
OIM Axial	UC at 1 month	18.04 ± 2.78	18.9 (15.9–20.2)	5.532	0.237
	UC at 3 months	18.06 ± 2.58	17.75 (16.4–19.3)		
	UC at 6 months	18.79 ± 2.23	19.1 (16.4–20.3)		
	UC at 12 months	17.64 ± 1.74	17.3 (16.3–18.4)		
	UI at 12 months	16.77 ± 3.32	17.3 (13.3–19.2)		
Pubococcygeus muscle coronal	UC at 1 month	3.69 ± 0.69	3.55 (3.2–4)	1.616	0.806
	UC at 3 months	3.78 ± 0.91	3.7 (2.9–4.4)		
	UC at 6 months	3.52 ± 1.01	3.6 (2.9–4.1)		
	UC at 12 months	3.67 ± 0.88	3.6 (3.3–4.1)		
	UI at 12 months	3.39 ± 0.86	3.5 (2.7–3.8)		
Pubococcygeus muscle axial	UC at 1 month	3.75 ± 0.83	3.7 (3.1–4.2)	0.525 ‡	0.717
	UC at 3 months	3.56 ± 0.78	3.5 (3–4)		
	UC at 6 months	3.69 ± 1	3.4 (3–4.4)		
	UC at 12 months	3.47 ± 0.85	3.4 (2.8–3.9)		
	UI at 12 months	3.79 ± 0.52	3.75 (3.6–4.2)		

* *p* < 0.05 and ‡ ANOVA test. UC: urinary continence, UI: urinary incontinence, MUL: membranous urethral length, MUW: membranous urethral width, MUA: membranous urethral angle, LAM: levator ani muscle, OIM: obturator internus muscle.

**Table 4:** Cox regression for time to continence (HR > 1 indicates faster continence recovery).

Parameter	Univariable HR (95% CI)	*p*	Multivariable HR (95% CI)	*p*
Age (per 5 years)	0.86 (0.76–0.98)	0.024	0.85 (0.74–0.98)	0.024
Prostate volume (per 10 cm^3^)	1.00 (0.95–1.05)	0.951	–	–
MUL (per mm)	1.04 (0.97–1.12)	0.252	1.05 (0.97–1.13)	0.250
MUW (per mm)	1.06 (0.95–1.18)	0.326	–	–
MUA (per 10°)	1.44 (1.23–1.68)	<0.001	1.43 (1.23–1.67)	<0.001
LAM coronal (per mm)	1.51 (1.32–1.73)	<0.001	1.30 (1.11–1.54)	0.002
LAM axial (per mm)	1.48 (1.30–1.68)	<0.001	1.28 (1.08–1.52)	0.004
OIM coronal (per mm)	1.09 (1.01–1.19)	0.033	–	–
OIM axial (per mm)	1.06 (0.98–1.14)	0.147	–	–
Pubococcygeus muscle coronal (per mm)	1.11 (0.90–1.38)	0.327	–	–
Pubococcygeus muscle axial (per mm)	1.02 (0.81–1.29)	0.840	–	–

The multivariable model included age, MUL, MUA, and coronal and axial LAM thickness. HR: hazard ratio; CI: confidence interval; MUL: membranous urethral length; MUW: membranous urethral width; MUA: membranous urethral angle; LAM: levator ani muscle; OIM: obturator internus muscle.

**Table 5:** (A) Multivariable logistic regression for UI at 3 months (54 events). (B) Univariable Firth logistic regression for UI at 12 months (10 events).

Parameter	OR (95% CI)	*p*
**A. UI at 3 months (multivariable logistic regression; 54 events)**		
Age (per 5 years)	1.39 (0.97–2.01)	0.077
MUA (per 10°)	0.48 (0.30–0.74)	0.001
LAM coronal (per mm)	0.57 (0.38–0.85)	0.005
LAM axial (per mm)	0.64 (0.42–0.96)	0.033
**B. UI at 12 months (univariable Firth logistic regression; 10 events)**		
Age (per 5 years)	1.46 (0.87–2.43)	0.151
MUA (per 10°)	0.23 (0.10–0.54)	<0.001
LAM coronal (per mm)	0.41 (0.23–0.76)	0.004
LAM axial (per mm)	0.42 (0.24–0.74)	0.003
MUL (per mm)	0.65 (0.45–0.94)	0.023

OR < 1 indicates lower odds of UI. Panel A: multivariable logistic regression; panel B: univariable Firth penalized logistic regression with Wald-type 95% confidence intervals. OR: odds ratio; CI: confidence interval; UI: urinary incontinence; MUA: membranous urethral angle; LAM: levator ani muscle; MUL: membranous urethral length.

## Data Availability

All datasets generated and analyzed during this study are available from the corresponding author on reasonable request and in accordance with institutional data sharing policies.

## Supplementary Materials

The following supporting information can be downloaded at: https://www.mdpi.com/article/10.3390/diagnostics16193203/s1, Figure S1: Calibration plot of the multivariable logistic model for urinary incontinence at 3 months.

## Abbreviations

The following abbreviations are used in this manuscript:

PSA	Prostate-specific antigen
RARP	Robot-assisted radical prostatectomy
UI	Urinary incontinence
UC	Urinary continence
MRI	Magnetic resonance imaging
LAM	Levator ani muscle
OIM	Obturator internus muscle
MUW	Membranous urethral width
MUL	Membranous urethral length
MUA	Membranous urethral angle
HR	Hazard ratio
CI	Confidence interval
GEE	Generalized estimating equations
AUC	Area under the receiver operating characteristic curve
BMI	Body mass index
OR	Odds ratio
ICIQ-UI SF	International Consultation on Incontinence Questionnaire–Urinary Incontinence Short Form
IIEF-5	International Index of Erectile Function-5
PLND	Pelvic lymph node dissection

## References

[1] Sung H., Ferlay J., Siegel R.L., Laversanne M., Soerjomataram I., Jemal A., Bray F. (2021). Global Cancer Statistics 2020: GLOBOCAN Estimates of Incidence and Mortality Worldwide for 36 Cancers in 185 Countries. CA Cancer J. Clin..

[2] Ficarra V., Novara G., Rosen R.C., Artibani W., Carroll P.R., Costello A., Menon M., Montorsi F., Patel V.R., Stolzenburg J.U. (2012). Systematic review and meta-analysis of studies reporting urinary continence recovery after robot-assisted radical prostatectomy. Eur. Urol..

[3] Marimpietri F.S., Tourinho-Barbosa R., Roldan-Testillano R., Batista L.T., Sanchez-Salas R. (2025). Lower urinary tract symptoms after robot-assisted radical prostatectomy: A systematic review. J. Robot. Surg..

[4] Kim L.H.C., Patel A., Kinsella N., Sharabiani M.T.A., Ap Dafydd D., Cahill D. (2020). Association Between Preoperative Magnetic Resonance Imaging-based Urethral Parameters and Continence Recovery Following Robot-assisted Radical Prostatectomy. Eur. Urol. Focus..

[5] Nakane A., Kubota H., Noda Y., Takeda T., Hirose Y., Okada A., Mizuno K., Kawai N., Tozawa K., Hayashi Y. (2019). Improvement in early urinary continence recovery after robotic-assisted radical prostatectomy based on postoperative pelvic anatomic features: A retrospective review. BMC Urol..

[6] Hashimoto S., Obinata D., Uchida H., Arakawa S., Inagaki Y., Nakahara K., Yoshizawa T., Mochida J., Yamaguchi K., Takahashi S. (2025). Preoperative pelvic floor muscle diameter as a predictor of postoperative urinary incontinence in robotic-assisted laparoscopic total prostatectomy. BJUI Compass.

[7] Gandaglia G., Ploussard G., Valerio M., Mattei A., Fiori C., Fossati N., Stabile A., Beauval J.B., Malavaud B., Roumiguié M. (2019). A Novel Nomogram to Identify Candidates for Extended Pelvic Lymph Node Dissection Among Patients with Clinically Localized Prostate Cancer Diagnosed with Magnetic Resonance Imaging-targeted and Systematic Biopsies. Eur. Urol..

[8] Sauer M., Tennstedt P., Berliner C., Well L., Huland H., Budäus L., Adam G., Beyersdorff D. (2019). Predictors of short and long term urinary incontinence after radical prostatectomy in prostate MRI: Significance and reliability of standardized measurements. Eur. J. Radiol..

[9] Kojima Y., Takahashi N., Haga N., Nomiya M., Yanagida T., Ishibashi K., Aikawa K., Lee D.I. (2013). Urinary incontinence after robot-assisted radical prostatectomy: Pathophysiology and intraoperative techniques to improve surgical outcome. Int. J. Urol..

[10] Al Kalailah F.M., Gu D., Wang Y., Li M., Zeng G. (2025). Short-term urinary continence outcomes after extraperitoneal single-site robot-assisted radical prostatectomy: A retrospective study. Asian J. Urol..

[11] Greco K.A., Meeks J.J., Wu S., Nadler R.B. (2009). Robot-assisted radical prostatectomy in men aged > or =70 years. BJU Int..

[12] Liu Y., Liu Z., Wang Z., Shao Y., Yang Z., Huang H., Wang Z., Fu Z., Wen S., Niu Y. (2025). The impact of prostate volume on Retzius-sparing robot-assisted laparoscopic radical prostatectomy with retrograde release of the neurovascular bundle. BMC Urol..

[13] Mac Curtain B.M., Sugrue D.D., Qian W., O’Callaghan M., Davis N.F. (2024). Membranous urethral length and urinary incontinence following robot-assisted radical prostatectomy: A systematic review and meta-analysis. BJU Int..

[14] Wang M., Deng R., Wang L., Li M., Zeng T., Na Y., Gu D. (2024). Association between 3D membranous urethral parameters and urinary continence recovery after RARP. Eur. J. Med. Res..

[15] Veccia A., Bertolo R., Artoni F., Bianchi A., De Marco V., Gozzo A., Migliorini F., Porcaro A.B., Raiti A., Rizzetto R. (2024). Surgical experience overcomes the impact of prostatic-urethral anatomy on continence recovery after robotic prostatectomy: Comprehensive analysis on 366 cases. World J. Urol..

[16] Tutolo M., Rosiello G., Stabile G., Tasso G., Oreggia D., De Wever L., De Ridder D., Pellegrino A., Esposito A., De Cobelli F. (2022). The key role of levator ani thickness for early urinary continence recovery in patients undergoing robot-assisted radical prostatectomy: A multi-institutional study. Neurourol. Urodyn..

[17] Tienza A., Hevia M., Benito A., Pascual J.I., Zudaire J.J., Robles J.E. (2015). MRI factors to predict urinary incontinence after retropubic/laparoscopic radical prostatectomy. Int. Urol. Nephrol..

[18] Li Y., Li W., Lu W., Chen M., Gao J., Yang Y., Zhuang J., Li X., Guo H., Qiu X. (2020). Association of preoperative urethral parameters on magnetic resonance imaging and immediate recovery of continence following Retzius-sparing robot-assisted radical prostatectomy. Transl. Androl. Urol..

