# Supplementary material for: Association of Preoperative MRI-Derived Anatomical Parameters with Urinary Continence Recovery After Robot-Assisted Radical Prostatectomy
Source: Diagnostics (Basel). 2026 Oct 2;16(19):3203. doi: 10.3390/diagnostics16193203 (PMC13664614; doi:10.3390/diagnostics16193203)
Supplement: Supplementary file 1 [file diagnostics-16-03203-s001.zip › diagnostics-4564442-supplementary.pdf]

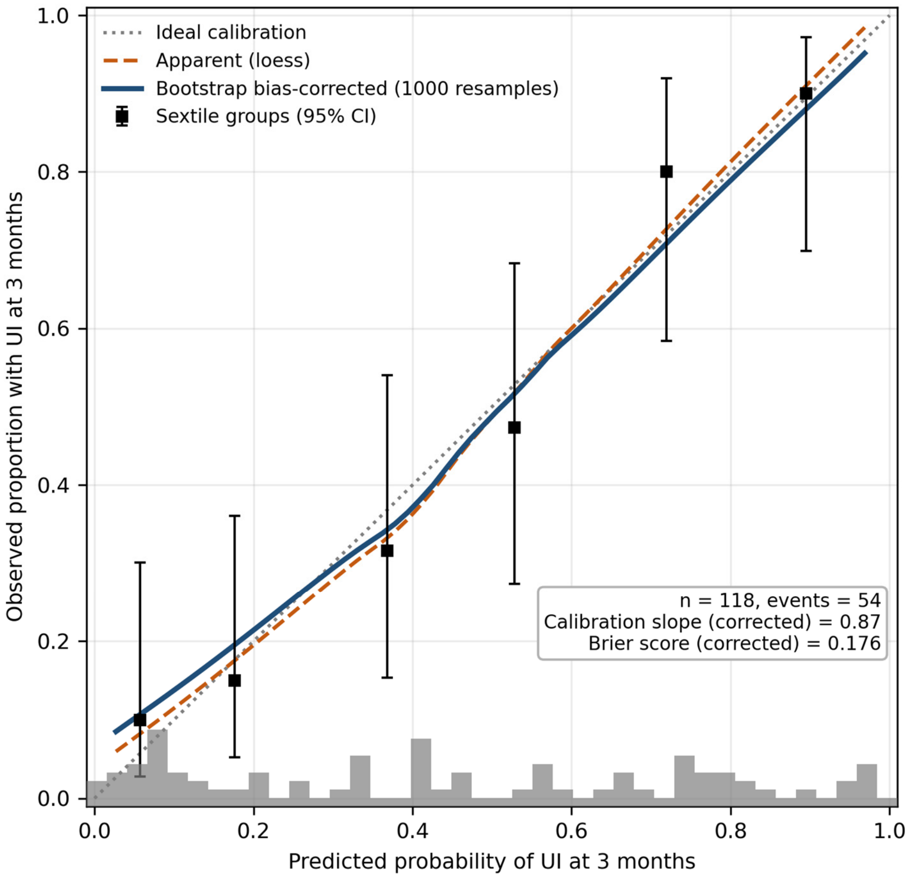

Figure S1. Calibration of the multivariable logistic model for urinary incontinence (UI) at 3 months (age, MUA, and coronal and axial LAM thickness; 54 events in 118 patients). The solid blue line shows the bootstrap bias-corrected calibration curve (1000 resamples, locally weighted smoothing), the dashed orange line the apparent calibration curve, and the dotted line ideal calibration. Squares show observed proportions with Wilson 95% confidence intervals in six groups of predicted risk. The histogram at the bottom shows the distribution of predicted probabilities. The bootstrap-corrected calibration slope was 0.87, and the bootstrap-corrected Brier score was 0.176 (0.248 for a model without predictors). MUA: membranous urethral angle; LAM: levator ani muscle.
